# Interdependent Effect of Intrinsic Risk Factors on Non-Contact Lower Limb Injuries in Male Football Players: A Structural Equation Modeling Approach

**DOI:** 10.3390/medicina62010052

**Published:** 2025-12-26

**Authors:** Nikolaos I. Liveris, Charis Tsarbou, George Papageorgiou, Elias Tsepis, Sofia A. Xergia

**Affiliations:** 1 Department of Physiotherapy, School of Health Rehabilitation Sciences, University of Patras, 26504 Rio, Greece; ctsarmpou@upatras.gr (C.T.); tsepis@upatras.gr (E.T.); 2 Systema Research Center, European University Cyprus, P.O. Box 22006, 1516 Nicosia, Cyprus; g.papageorgiou@euc.ac.cy

**Keywords:** sports injury, prevention strategies, functional assessment, injury risk factors interrelationships, lower limb injuries

## Abstract

*Background and Objectives*: Recent research has highlighted the importance of examining risk factors and their complex interrelationships in the development of lower limb injuries. This study aimed to investigate the direct, indirect, and mediating effects of endogenous neuromuscular and psychological risk factors on the incidence of non-contact lower limb injuries in football players. *Materials and Methods*: A sample of ninety-seven male football players underwent a structured preseason, on-field assessment. Demographic characteristics, injury history, and athlete burnout were collected through standardized questionnaires. Preseason evaluations included assessments of lower limb flexibility; isometric strength assessment of hamstring, hip abductors, and quadriceps using a handheld dynamometer; hamstring and core endurance; and the single-leg triple hop for distance test. All non-contact lower limb injuries were prospectively recorded throughout the competitive season. Partial Least Squares Structural Equation Modeling (PLS-SEM) method was applied to examine both direct and indirect associations between preseason risk factors and injury incidence. *Results*: Lower limb strength asymmetries (path coefficient (PC) 0.293, *p* = 0.004) and previous injuries (PC 0.233, *p* = 0.015) exhibited the strongest direct effects on the occurrence of new non-contact lower limb injuries. In addition, age acted as a moderating factor, amplifying the effect of lower limb strength asymmetries on injury risk. Moreover, previous injuries demonstrated both direct and indirect effects on neuromuscular characteristics and perceived burnout. Core and hamstring endurance tended to influence new injuries indirectly through strength asymmetries and were significantly affected by hamstring strength (PC 0.248, *p* = 0.015) and prior injuries (PC −0.207, *p* = 0.029). *Conclusions*: Injury prevention strategies should prioritize the improvement of core and hamstring endurance and the reduction in lower limb Strength Asymmetries, particularly among older football players. Furthermore, individualized preventive interventions for athletes with a previous history of injury are strongly recommended.

## 1. Introduction

Football is one of the most demanding team sports, requiring high-intensity intermittent sporting activities such as accelerations, decelerations, jump landing, and rapid change of directions [1,2]. These sporting movements result in increased physiological demand of the athlete’s musculoskeletal tissues, thereby elevating injury risk [1]. Consequently, football has increased incidence of non-contact lower limb (LL) injuries, which have a significant impact on athletes’ well-being and athletic performance [2,3,4]. Sports injuries are more frequent during matches than training with a total incidence of up to 51.4 injuries per 1000 h of match exposure compared to 3.81 injuries per 1000 h of football training exposure [1]. Severe knee injuries [5], including Anterior Cruciate Ligament (ACL) injuries [6], severe ankle sprains [7], and hamstring injuries [8], are highly prevalent in football.

A large number of studies have investigated injury patterns and the multiple risk factors in sport injury etiology [1,2,9]. Factors such as movement biomechanics, fatigue, and workload formulate a complex network of factors influencing injury occurrence [10,11,12]. Despite this extensive body of research, the relative contribution and interaction of these risk factors remain unclear. Based on the recent literature, this uncertainty is attributed to the complex nature of sports injury [11,13]. Complex system theory suggests that sports injury risk factors interact dynamically, with many exerting indirect rather than direct effects on injury outcomes [10,11]. In contrast, traditional research approaches tend to examine individual risk factors in isolation, without accounting for the dynamic and independent relationships among risk factors [3,4,13]. Utilizing this approach, most studies in the sports injury field employ a prospective cohort design, wherein multiple risk factors are assessed during the preseason preparatory phase, and injury incidence is prospectively monitored throughout the competitive season [11]. Subsequently, researchers attempt to identify isolated associations between the independent variables (risk factors) and the dependent variable (injury) by applying various regression-based statistic models [14]. While this methodology can identify linear and robust predictors for injury, it fails to capture the complex indirect effect through which certain risk factors may influence injury risk [13].

To evaluate and quantify potential direct and indirect effect of risk factors on injury occurrence, various statistical and simulation modeling approaches have been proposed and applied within the field of sports injury research. Machine learning techniques have gained popularity, as they enable the nonlinear evaluation of large datasets comprising multiple risk factors and injury outcomes, such as thigh muscle injuries [15] and hamstring injuries [16,17]. Moreover, alternative modeling approaches including Agent-Based Modeling [18] and qualitative causal loop modeling have been employed to capture the complex dynamics underlying sports injuries [3,4]. These multivariate procedures have provided essential contributions to injury prediction and the visualization of system complexity [3,19]. However, these methods have limited capacity to simultaneously quantify and statistically test hypothesized direct and indirect causal relationships among multiple interrelated risk factors [19]. Structural Equation Modeling (SEM) can fill this gap by providing a comprehensive framework to evaluate complex causal pathways underlying sports injury risk [20].

Particularly, SEM constitutes a powerful multivariate statistical methodology that allows for the quantification of complex direct and indirect interrelationships among multiple observed variables [20]. SEM encompasses a range of multivariate statistical techniques for data analysis within a single integrative analytical framework, including exploratory factor analysis, which groups measured variables into latent constructs, confirmatory factor analysis, and multiple regression analysis [21]. A distinctive capability of SEM is its ability to construct latent—unobserved factors based on the increased correlation of different measured—observed variables which reflect the latent factor [20]. Accordingly, SEM facilitates the examination of complex causal models, including direct indirect/mediating, and moderating relationships among latent constructs and allows for the simultaneous testing of multiple hypotheses [21]. In addition, latent constructs within SEM can function as both independent and dependent variables within the same model, thereby overcoming a key limitation of traditional linear statistical approaches such as linear regression, which typically assess each relationship in isolation [20]. These advantages of SEM support the development and testing of theory-driven models, thereby enhancing the understanding of the complex multifactorial nature of sports injury. As such, SEM provides robust capabilities for exploration and confirming theoretical models by quantifying latent factors’ interrelationships. These capabilities are unique in SEM in contrast to other multivariate approaches, such as machine learning, which mainly focus on classification and prediction based on complex patterns of large datasets [19]. However, despite these extensive capabilities of SEM, only few studies, in the field of sports injury, have utilized this statistical approach [22]. The application of SEM in the sports injury domain may enhance the understanding of the complex indirect and moderating effects of risk factors on injury.

Therefore, the primary aim of the current study is to evaluate the interrelationships of multiple intrinsic risk factors and their direct, indirect, and mediating effects on the incidence of non-contact LL injuries in football players using SEM analysis.

### Structural Hypotheses

**Figure 1:**
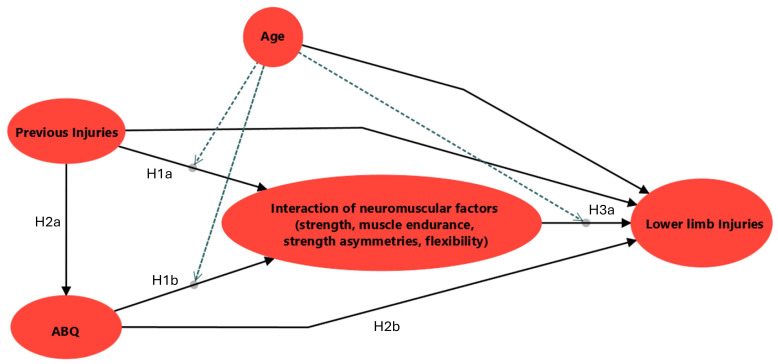
Proposed conceptual model and structural hypotheses. Abbreviations: ABQ—athlete burnout questionnaire items; H—hypothesis.

The previous literature and qualitative analyses of our research team [3,4,10] provide evidence for the interrelationships among injury risk factors and were used as the bases for formulating specific structural hypotheses as illustrated in Figure 1. These hypotheses were categorized into direct, indirect/mediating, and moderating effects. Specifically, strong evidence from the literature supports the effect of previous injuries on the occurrence of subsequent injury [23]. Based on this evidence, we hypothesized that previous injuries influence the risk of new injuries either directly (Figure 1, H1a) or indirectly (Figure 1, H2a) through the negative impact on neuromuscular characteristics including muscle strength, muscle endurance, strength asymmetries, and flexibility [23]. In addition, previous studies [15,17] have reported a potential association between burnout-related parameters, as assessed by using the athlete burnout questionnaire (ABQ), and the occurrence of new injuries. These findings suggest that burnout may be influenced by both the quality of previous injury and athletes’ neuromuscular capacities [15,17]. Accordingly, we hypothesized that a greater number of previous injuries would be associated with higher ABQ scores [15,17] (Figure 1, H2a). Furthermore, higher ABQ scores were hypothesized to influence injury risk both directly and indirectly via their effect on neuromuscular quality, including strength, endurance, and asymmetry-related factors (Figure 1, H1b and H2b). Although association between ABQ, previous injuries, and neuromuscular abilities has been previously documented, the magnitude and direction of the direct effects of burnout on neuromuscular coordination remain unclear [15,17]. Moreover, neuromuscular factors such as lower limb strength, hamstrings and core endurance, and strength asymmetries were hypothesized to be interrelated and may be modeled as distinct latent constructs within the SEM framework [3,4]. Likewise, reduced neuromuscular quality, reflected by lower strength, decreased muscular endurance, greater strength asymmetries, and reduced flexibility, was hypothesized to be associated with a higher likelihood of non-contact LL injuries (Figure 1, H3a). Finally, age, previous injuries, and burnout (ABQ), as factors influencing athlete’s internal characteristics, were hypothesized to act as moderators of selected interrelationships within the proposed model [3,15].

## 2. Materials and Methods

### 2.1. Study Design

This study adopted a prospective cohort design to examine the interrelationships among various intrinsic risk factors and their effects on non-contact LL injuries in football players. During the 2022–2023 competitive season football players were evaluated in the first two weeks of the preseason phase using a standardized field-based screening protocol. Then, participants were prospectively followed throughout the competitive season to record all time-loss non-contact LL injuries. This investigation forms part of a larger research project, with the full study protocol registered at ClinicalTrials.gov (ID: NCT05425303) and approved by the Institutional Ethics Committee of the University of Patras. Additionally, this study was reported in accordance with the Strengthening the Reporting of Observational Studies in Epidemiology (STROBE) guidelines [24].

### 2.2. Participants

**Figure 2:**
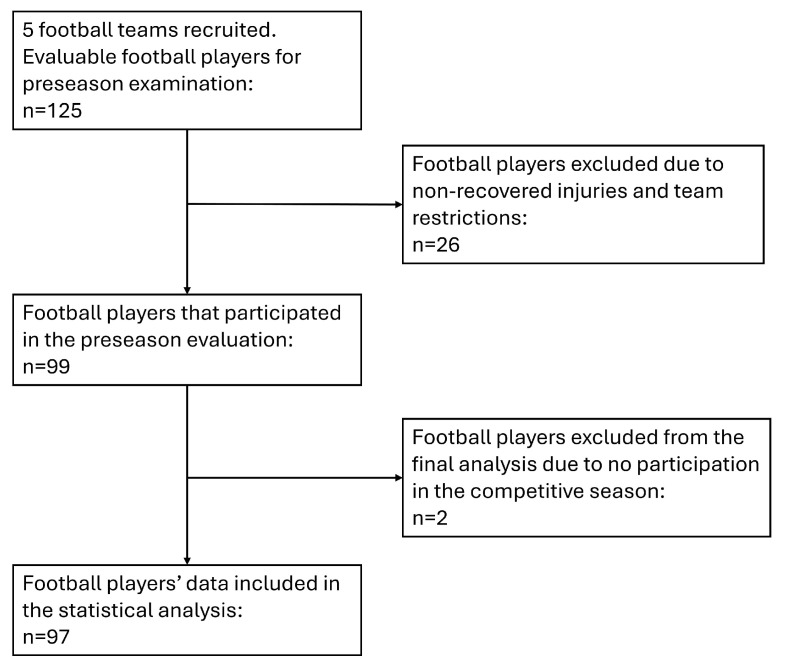
Flowchart of participants’ inclusion in the study.

Football players from five teams were recruited to participate in this study [mean, standard deviation (SD), age 22.16 (5.03); weight 74.44 (7.71) kg; height 178.82 (6.27) cm; BMI 23.25 (1.76)]. A convenient sampling approach was employed, comprising teams that provided consent to participate. The sample included three teams from the second division of Greek football, one team from the third division, and one under-19 division. Out of one hundred and twenty-five eligible football players, ninety-nine participated in the preseason assessment. The remaining football players were excluded due to recent injuries, team-imposed scheduling constraints, or incomplete measurements caused from pain or joint instability. The eligibility criteria required that participants be either injury-free or fully recovered from any previous injury, have returned to full participation in football following medical clearance from the team physician [25], hold a professional contract with their respective teams, and have engaged in five to six training sessions per week during the previous season, with minor adjustments based on match scheduling. In addition, two participants retired and did not participate in the competitive season and were therefore excluded from the final analysis. Consequently, data from ninety-seven football players were included in the analysis. Participants’ characteristics are summarized in Table 1, and the flow of participants’ inclusion is illustrated in Figure 2.

An a priori power analysis was conducted to determine the minimum sample size required for Partial Least Squares Structural Equation Modeling (PLS-SEM) based on the most complex regression in the structural model, which indicates the endogenous construct with the largest number of predictors [26]. Using G*Power (version 3.1.9.7) for a multiple linear regression (fixed model, R^2^ deviation from zero), the analysis indicated that a minimum sample size of 98 participants was required to detect a medium effect size (f^2^ = 0.15) with an alpha level of 0.05 and a statistical power of 0.80.

### 2.3. Preseason Data Collection

**Figure 3:**
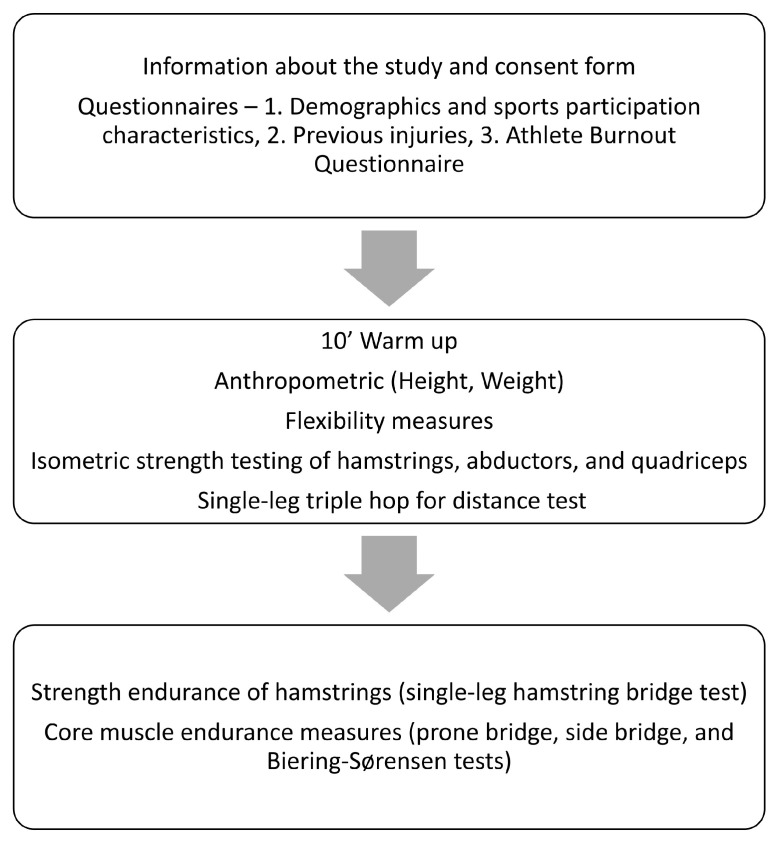
Measurement process.

The current measurement protocol is part of a more holistic evaluation, and data have been used for various studies, which have been reported in previous papers [27,28]. Below, we present the measurements and data used for the current analysis. Initially, participants read and signed the consent form and then completed three specific questionnaires. Afterwards, the data collection of internal neuromuscular characteristics was carried out using field-based clinical measures as presented in the following subsections. The measurements process is presented in Figure 3. Each participant required approximately ninety minutes to complete the screening protocol. The entire evaluation was conducted at the teams’ facilities, utilizing portable low-cost equipment.

#### 2.3.1. Survey Questionnaires

At the beginning of the preseason evaluation, participants completed three questionnaires collecting information for football players’ (1) demographic and sports participation characteristics of previous years, (2) previous injury characteristics, and (3) their sense of burnout. One of the examiners assisted participants in filling out the questionnaires. As regards the collection of previous injury characteristics, a specific questionnaire, based on reported guidelines and previously published studies, was utilized to gather information about previous injuries [25,29,30]. The collected data included the number of previous injuries, injury type, limb of injury, time of injury, and time loss from sports participation due to injury. Additionally, participants reported the sense of burnout using the Greek version of the ABQ [31,32]. The Greek adaptation of the ABQ consists of 13 questions related to three aspects of athletes’ burnout. Five questions related to the assessment of “physical–emotional exhaustion”, five questions assess the “reduced sense of accomplishment”, and three questions assess the “sports devaluation”. Responses were given on a 5-point Likert scale, ranging from “almost never” (1) to “almost always” (5) [32]. For athletes who were not Greek, comprising less than 10% of the participants, the English version of the questionnaires was provided.

#### 2.3.2. Screening Tests

After completing the questionnaires, the athletes engaged in a ten-minute football-specific warm-up, which included running, agility exercises, and stretching. Afterward, a detailed field-based examination protocol was applied to evaluate various athletes’ physiological characteristics. Precisely, data collected included anthropometric characteristics (height and weight), lower limb flexibility and range of motion, thigh muscles strength, ballistic function using the single-leg triple hop for distance (THD) test, hamstring strength endurance, and core muscle endurance. Weight was collected using data for a static measurement on a force platform [40 × 60 cm, (Bertec, Columbus, OH, USA), sampling frequency of 1000 Hz] used for the purpose of a concurrent study that examined the participants’ landing performance [28]. Three evaluators conducted all assessments, each focusing on a distinct component of the screening protocol. Specifically, one evaluator was responsible for initially informing the participants, administering questionnaires, and collecting anthropometric data. Another evaluator handled the measurements of flexibility and isometric strength testing. The third evaluator conducted the THD test and assessed hamstring and core muscle endurance. All evaluators were physical therapists, with two being PhD candidates and one an MSc student, each possessing 5–10 years of clinical experience. Prior to this study, pilot applications were carried out to evaluate the protocol’s implementation and to familiarize the evaluators with the measurement procedures.

Details about the tests used during the preseason field-based evaluation are presented in Table 2.

Following questionnaire supplementation, warm-up, and anthropometric measurements, participants underwent a comprehensive passive lower limb flexibility evaluation. Particularly, the flexibility of hamstrings, iliopsoas, rectus femoris, hip internal–external rotation, and ankle dorsiflexion were evaluated using a portable bubble inclinometer (Baseline, New York, NY, USA) [33]. From the supine position, the passive straight leg raise test was employed to assess hamstring flexibility as previously reported [34,35]. From the same position, the Thomas test was applied to evaluate Iliopsoas flexibility. The Thomas test was qualitatively evaluated as pass or fail in accordance with the guidelines established by Peeler and Anderson [36]. In a positive Thomas test, knee flexion was observed while the contralateral lower limb was flexed on the participant’s chest [36]. Moreover, from prone position, Ely’s test [37] was conducted to assess the rectus femoris’ flexibility. For the same position, the range of motion of hip internal and external rotation were measured. From 90° of knee flexion, the examiner moved the lower limb to the internal or external hip rotation to observe the range of motion, taking care to prevent pelvic motion [35]. In addition, for the upright position, ankle dorsiflexion was evaluated using the weight-bearing lunge test [38,39]. The measure was obtained by positioning the inclinometer at the upper third of the tibia at the end of the range of motion. Two measurements were executed for all flexibility evaluations, and an additional measure was performed in case of >5% difference between the first two records. To receive each flexibility measure, the following criteria were applied: (1) the participants should experience stretching but not pain, (2) the examiner should encounter resistance against their movement, and (3) the flexibility measure should be recorded immediately before the onset of additional movements of the peripheral joints or pelvis [33,35]. The average value for each flexibility test was used for statistical analysis.

After the flexibility tests, participants were subjected to isometric strength testing of the hamstring [40,41], abductors [42], and quadriceps muscles [43] following the corresponding reported guidelines for each test. In the case of the hamstring strength examination, the hold test (isometric) and brake test (eccentric) were utilized [40,41]. Isometric strength testing using a handheld dynamometer (HHD) provides an acceptable method for field-based evaluation, as it has been proven to have moderate to good validity and reliability in relation to the isokinetic dynamometer [44]. In all muscle group examinations, participants performed two familiarization submaximal muscle contractions for approximately 2 s. Subsequently, three maximal voluntary contractions lasting approximately 5 s, separated by a rest of at least 20 s, were performed for each lower limb. The higher value recorded in Newton (N) was used for statistical analysis and computation of limb symmetry. Particularly, all absolute strength values, multiplied by the lever arm, normalized to body weight, and expressed as Nm/kg [45,46,47,48]. All strength measurements were performed by the same examiner using an HHD (MicroFET 2; Hoggan Scientific, Salt Lake City, UT, USA). Stabilization of the athletes during strength testing was performed by one of the examiners using a stabilization belt. The detailed protocol of muscle strength evaluation is presented in previously published papers for our research group that present different analyses using the same data [27,28,49].

Following the strength measures, participants had two to three minutes of rest, and then the THD test was used to evaluate the participants’ ballistic performance. THD has acceptable test–retest reliability (ICC = 0.91) [50] and represents a functional evaluation of lower limb strength and power [50,51,52]. After two familiarization attempts, three successful trials for each lower limb were recorded. The participants needed to maintain their balance during the final landing for at least two seconds to deem a trial as successful. To prevent fatigue, participants altered the lower limb in each trial, and a period of rest of approximately 30 s was allowed between trials. The hop distance in centimeters was recorded. The highest measured distance was divided by the participant’s height to produce a normalized value (maximum distance in cm/athlete’s height), which was used for the statistical analysis [52,53].

Endurance measures were performed at the end of the screening protocol to prevent possible fatigue effect on other measurements. These measures included the single-leg hamstring bridge (SLHB) test for the evaluation of the hamstring endurance ability [54], as well as the evaluation of core muscles endurance via prone bridge, side bridge, and Biering–Sørensen tests. After a resting period of approximately three to five minutes from the other measurements, participants performed the endurance tests in random order. For the proper execution of the SLHB test, previously reported guidelines [54] were followed. The participants performed single-leg pelvis raising until failure due to fatigue. The test configuration required the participant to raise the pelvis up to 0 degrees of hip flexion and then lower it until touching the ground without resting. The testing leg should be on a 60 cm box, the untested leg flexed, and the upper limbs crossed to the chest. The total number of repetitions was used for statistical analysis as proposed in a previous investigation [54]. To measure the endurance ability of abdominal muscles, the prone bridge [55] and the side bridge tests [56] were utilized for the rectus and lateral abdominal muscles evaluation, respectively. The prone bridge has proven to be a valid and reliable tool for rectus abdominal endurance evaluation, and the reported guidelines were followed [55]. As regards testing of lateral abdominals, the side bridge test was performed bilaterally following the reported guidelines [56]. Moreover, the endurance ability of core extensors was evaluated by the execution of the Biering-Sørensen test based on the reported guidelines [56,57]. The Biering–Sorensen test is a valid clinical test for back muscle endurance evaluation [57]. The core endurance tests require the participants to hold the appropriate position for as much time as they can until failure due to fatigue. The holding time was manually recorded in seconds, and the maximum time was used for statistical analysis. Initially, the examiners informed the participants for the proper execution of the tests, and a familiarization submaximal trial was allowed. During the execution of the tests, one examiner carefully reviewed the proper position of participants and motivated participants to hold for as much time as they can. Participants received one warning for incorrect positioning; the test was terminated after a second warning or when the correct position could no longer be maintained because of fatigue [55,56,57].

### 2.4. Injury Surveillance

Each team’s medical staff had a designated member responsible for recording all injuries daily. The principal investigator gathered information on all time-loss injuries every two weeks by communicating with the medical practitioner of each team. Injuries were collected starting from the day of baseline measurements and continued to be recorded throughout the entire 10-month competitive season. The injury data covered the injury type, mechanism of injury (contact, non-contact), time loss from participation in football training and competition, date of injury occurrence, date of full return to competition, and the affected lower limb. Based on the above-mentioned information, the principal investigator (NIL) distinguished between contact and non-contact injuries. An injury was defined in practical terms as any physical issue encountered by the athlete while playing football that hindered their usual physical abilities, leading to an inability to fully engage in football activities for at least one day [25]. Lower limb injuries were specified after physical examination and diagnostic imaging by the team’s physician. The frequency of total-recorded non-contact LL injuries for each football player and the time loss associated with these injuries were utilized for statistical analysis. The frequency of each type of injury was determined by calculating the total hours of training and match exposure for each team, represented as injuries per 1000 athlete-hours of exposure. The following formula used was: [(Number of injuries/total exposure to training and competition) × 1000] [25].

### 2.5. Data Processing and Statistical Analysis

Initially, data were inserted into Microsoft Excel for initial evaluation and basic variables calculations. Specifically, inter-limb symmetry of all bilateral strength and flexibility measures was estimated using the formula [(dominant-nondominant)/maximum (dominant or nondominant) × 100] [58]. In addition, strength values were multiplied by the lever arm and normalized to body weight to be expressed as Nm/kg [45,47,48]. Furthermore, the THD performance was normalized with the athlete’s height to provide body size-independent values, as previously described [52,53]. Regarding the previous injuries data, the number of previous injuries and the most recent previous injury in either the dominant or non-dominant limb, with the corresponding time loss, were the main variables used for statistical analysis. The aforementioned data for both LL were used as independent variables in the statistical analysis. The frequency of all non-contact lower limb injuries was used as the dependent variable.

Then, data were included in the SPSS (version 28) software for screening, descriptive statistics (mean and standard deviation), and exploratory factor analysis (EFA). Initially, data were screened for missing values, outliers, and normality. Missing values were below 5% of the data and were replaced with the median for ordinal variables and the mean for continuous scales [59], as this is an acceptable method when there is <5% missing data for each variable [59,60]. Additionally, data were assessed for outliers using boxplots and Mahalanobis distance to detect possible multivariate outliers. During this procedure, entry error outliers were adjusted, and an extreme outlier was considered as a missing value [59,60]. The normal distribution of the data was evaluated with skewness, kurtosis, and visually with histograms. A perfectly normal distribution is characterized by skewness and kurtosis values of zero. Nonetheless, it has been noted that skewness can reach up to 2.0 and kurtosis up to 7.0, and this criterion was followed in the current study [59]. Following the data screening, EFA was utilized for all independent variables using promax rotation and principal component analysis extraction methods. The purpose of EFA is to identify the grouping of the measured variables into latent factors based on the variables intercorrelations [59,61]. In this stage, variables that provide low loadings values (<0.5) in latent factors or high cross-loading among various latent factors were excluded from the analysis [27,59,61].

The PLS-SEM analysis was then utilized to explore the interdependences of latent factors, resulting from the EFA, and their effect on the frequency of non-contact lower limb injuries recorded during the competitive season. The SmartPLS software (4.1.0.6) was employed to perform the PLS-SEM analysis. The purpose of PLS-SEM is to evaluate the possible interrelationships of latent factors rather than confirm which is the main objective of covariance-based SEM. In addition, PLS-SEM has the advantage of providing solutions with smaller sample sizes and does not require strict data normality assumptions [20]. These make the PLS-SEM method appropriate for the purposes of the current study. PLS-SEM uses a series of ordinary least squares regression equations simultaneously to calculate the interrelationships among latent factors [26,62]. Τhe PLS-SEM was evaluated in terms of model fit, measurement model, and structural model.

During the model formulation, Standardized Root Mean Square Residual (SRMR) fit index was reviewed to evaluate the overall model fit. According to reported guidelines, a value < 0.1 is deemed acceptable [60,63].

The next step involved evaluation of the measurement (outer) model, which assesses how well each latent factor fits its indicators (measured variables) [26,62]. Evaluation of the measurement (outer) model considered indicator loadings on latent factors, along with each factor’s reliability and validity. Regarding indicators’ loading on each latent factor, a recommended threshold is that all indicators or the mean of the indicators’ loading should be above 0.708 [21,62]. Moreover, internal consistency reliability was evaluated based on the results of Cronbach’s Alpha, Reliability p_A_, Composite reliability p_c_. For these measures, a range of values between 0.60 and 0.90 is considered acceptable. The Average Variance Extracted (AVE) value was also evaluated for convergent validity. According to the reported guidelines, AVE above 0.50 [26,62]. In addition, the heterotrait–monotrait (HTMT) ratio of the correlation values was evaluated to ensure for the discriminant validity of the measurement model. Discriminant validity assesses the distinctiveness of a latent factor construct from other constructs within the model, and for this reason, HTMT values are recommended to be below 0.85 [26]. The above-mentioned criteria are essential to have a reliable and valid latent factor construct and affect the overall model fit [26]. Based on the measurement model evaluation, measurement indicators were excluded from the model if they did not fulfill the aforementioned requirements, affecting the reliability and validity of the latent factor construct [21].

**Figure 4:**
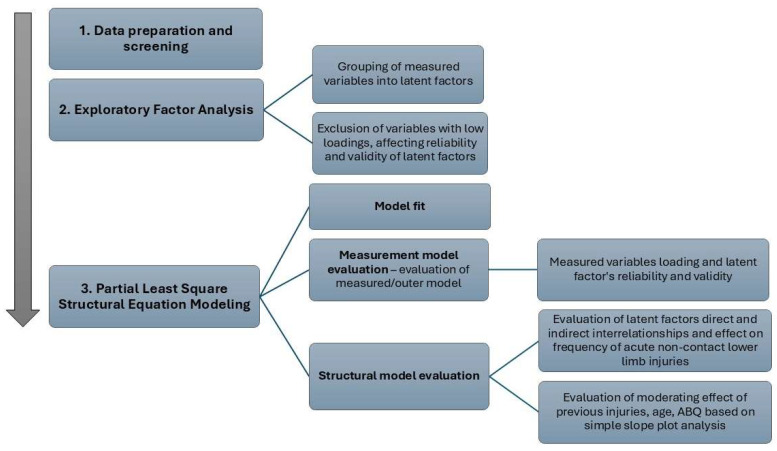
Data analysis workflow.

The assessment of the structural model includes the determination of the latent factor interrelationships and the explanatory power of the effect on each latent factor. Notable evaluated principles include the coefficient of determination (*R*^2^) values and the relevance and statistical significance of path coefficients. Variance inflation factor (VIF) values were initially assessed to ensure no collinearity issues, with values expected to be below 5 [26]. Then, R^2^ values of the endogenous latent factors were assessed to specify the explained variance of each endogenous latent factor, which is an indicator of the explanatory power of the model [26,62]. The R^2^ values ranged from 0 to 1, with values ≥ 0.75 considered substantial, ≥0.50 moderate, and ≥0.25 weak [62]. However, R^2^ values should be interpreted based on the specific context of each study, and for some fields, R^2^ values of 0.25 may be considered appropriate [62]. In the next step, we examined relevance and significance of the model relationships based on the path coefficient values. Path coefficients range from −1 to +1, and a higher value indicates a stronger positive (+) or negative (−) relationship. Path coefficients were interpreted based on *p*-values and t-statistics, which specify the statistical significance of the relationships, and were used as criteria for the evaluation of this study’s structural hypotheses. Relationships with *p*-value < 0.05, and t-statistics > 1.96, were considered significant [26,64]. In addition, effect size (f^2^) values were evaluated to interpret the strength of the relationships: values > 0.02 were considered small, >0.15 moderate, and >0.35 large effect sizes [26,62]. Interrelationships with extremely low f^2^ values were excluded from the model. In conclusion, we assessed the moderating effects of previous injuries, athletes’ burnout, and age on the model’s interrelationships through a simple slope plot analysis [26]. Figure 4 summarizes the aforementioned data analysis workflow.

## 3. Results

### 3.1. Descriptives of Preseason Screening Data

During the competitive season, all teams involved in the study adhered to a schedule, which included five to six training sessions and one football game per week.

For the sample included in the preseason examination, sixty-three participants (63.6%) reported one to three previous injuries. Among previously injured participants, most (38%) experienced a time loss of 8 to 28 days, followed by 1–3 months (30.1%) and more than 3 months (11.1%). For 93.9% of participants, the most recent previous injury occurred at least 6 months before the day of preseason measurements. All participants included in the preseason examination were fully recovered and returned to previous activity level without restrictions. The most frequently reported injuries were related to thigh, groin, or hip (17.2%), followed by knee injuries (other knee injuries 12.1%; ACL 5.1%) and ankle injuries (11.1%).

Table 3 summarizes the descriptive statistics of the preseason measurements. The variable “Devaluation” slightly exceeded the normality threshold based on skewness and kurtosis values. Nevertheless, as PLS-SEM does not require the assumption of normality, this variable was retained in the analysis.

### 3.2. Prospective Injury Data

**Figure 5:**
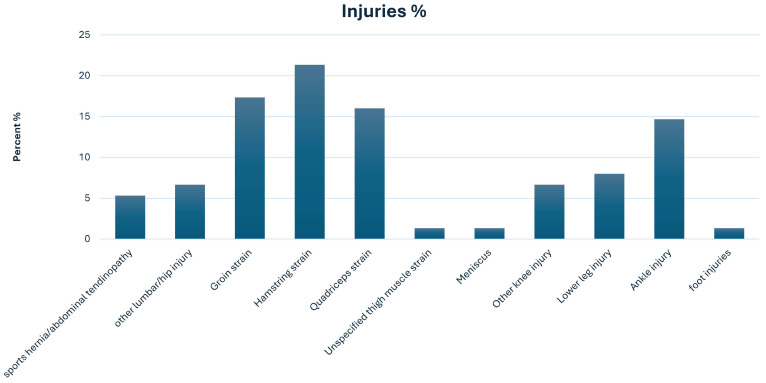
Frequency of the recorded lower limb injuries during the competitive season.

During the prospective evaluation, forty-three football players sustained up to five non-contact lower limb injuries during the competitive season. In total, seventy-five non-contact lower limb injuries and re-injuries were documented. Among those injuries, the hamstrings were the most common affected muscle, representing 21.3% of all injuries, followed by groin (17.3%), quadriceps (16%), and ankle (14.7%) injuries (Table 4 and Figure 5).

### 3.3. Exploratory Factor Analysis

EFA was initially performed to identify the underlying factors for the subsequent PLS-SEM analysis. The optimal factor solution accounted for 74.7% of the variance, yielding a Kaiser–Meyer–Olkin (KMO) measure of sampling adequacy of 0.7, with Bartlett’s test of sphericity demonstrating statistical significance at *p* < 0.001. Based on the eigenvalues and scree plot, the model resulted in seven latent factors, grouping twenty-one measured indicators. The latent factors model resulting from the EFA included the following components: (1) HS and Core Endurance: Comprised six measured items, including core and hamstring endurance measurements, (2) Hamstring Strength: Grouped the factors of isometric (make test) and eccentric (brake test) of both lower limbs, (3) ABQ: Contained the three components of ABQ-emotional and physical exhaustion, devaluation, and reduced sense of accomplishment, (4) Triple Hop for Distance: Grouped the THD measures of both LL, (5) Quadriceps Strength: Grouped quadriceps isometric strength measures of both lower limbs, (6) Previous Injuries: Comprised the variables of the number of previous injuries and the time loss of the most recent injury, and (7) Lower Limb Strength Asymmetries: Grouped the THD and hamstring isometric (make test) strength asymmetries.

In this stage, flexibility measures and other asymmetry measures were excluded for the analysis as they did not fulfill the requirements for inclusion into EFA and PLS-SEM analysis. Specifically, these variables resulted in low loadings and high cross-loadings among various latent factors and did not provide a clear solution, affecting the reliability and validity of the latent constructs.

### 3.4. PLS-SEM of Risk Factors Interrelationships That Affect the Frequency of Non-Contact LL Injuries

The results of EFA were used as input to introduce the main latent constructs in the PLS-SEM model. Nevertheless, during the PLS-SEM measurement model evaluation, latent factors of THD and quadriceps strength was grouped in the same latent construct, providing appropriate loading and validity–reliability values. Subsequently, the interrelationships of six latent factors and their effect on the Number of New Non-contact LL Injuries were analyzed in PLS-SEM. The model achieved an acceptable fit based on the SRMR results (saturated model 0.091, estimated model 0.093). The evaluation of measurement model, structural model, and moderators’ analysis are described below.

#### 3.4.1. Measurement Model Evaluation

The measurement model was evaluated based on the criteria of measured indicators’ loadings, latent factor validity, reliability, and discriminant validity. Measured indicators have proper loadings in each latent factor, as most measured indicators were above the recommended threshold of 0.7. The only exceptions were the measured indicators of THD asymmetries (0.614) and the Biering–Sorensen test (0.571), which exhibited the lowest loading values. However, these did not compromise the reliability and validity of the latent construct. Consequently, all latent constructs proved to have an appropriate level of construct reliability and validity, as measured by the AVE, Cronbach’s Alpha, Reliability pA, and Composite Reliability pc. Notably, the latent factor of LL Strength Asymmetries exhibited a low Cronbach’s alpha value of 0.5. However, the other reliability and validity measures yielded satisfactory results.

Moreover, the discriminant validity of the measurement model [62] was sufficient, as all latent constructs exhibit HTMT values below the more conservative threshold of 0.85, also considering the 95% confidence interval [26]. Table 5 delineates the principal measures of the measurement model.

#### 3.4.2. Structural Model Evaluation

**Figure 6:**
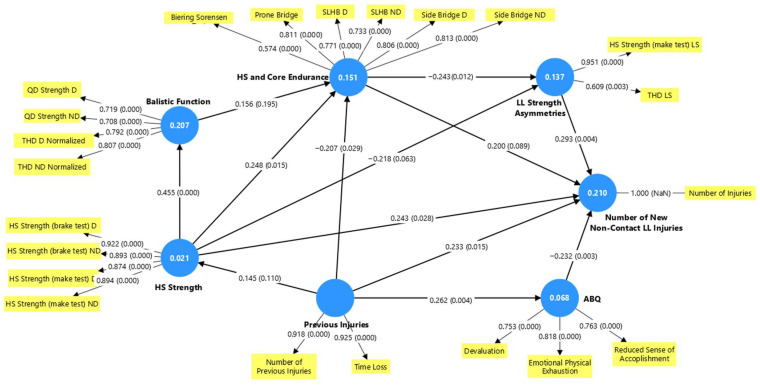
Structural model results. Path coefficients and *p* values of direct effects in parentheses. Abbreviations: ABQ—athlete burnout questionnaire, HS—hamstring, QD—quadriceps, LL—lower limb, D—dominant, ND—non-dominant, THD—single-leg triple hop for distance, SLHB—single-leg hamstring bridge, LS—limb symmetry.

Figure 6 illustrates the structural model results, showing the interrelationships among factors, the relevance and significance of each path (*p*-values for each path in parentheses), and the R^2^ values (shown in the circles) for all endogenous constructs. In addition, no collinearity issues were observed, as all latent factor constructs had VIF values below 3. The model explains 21% of the variance of the Number of New Non-contact lower limb injuries recorded during the competitive season.

All direct effects with confidence intervals are presented in Table 6. Table 7 presents the total effects that collectively consider the direct and indirect effects in the model, and Table 8 presents the total indirect effects created in the model. Nine paths had statistically significant direct effects in the model (Table 6). Regarding the relationships that agree with the study’s hypotheses, the model provides evidence for a direct significant positive relationship between increased LL Strength Asymmetries and the Number of New Non-contact LL Injuries (PC 0.293, *p* = 0.004). In addition, LL Strength Asymmetries were significantly and directly affected by Hamstring and Core Endurance (PC −0.243, *p* = 0.012) and demonstrated a near-significant relationship with HS Strength (PC −0.218, *p* = 0.063). These results indicate that as these factors increased, LL Strength Asymmetries decreased. HS Strength also had a statistically significant effect (PC 0.248, *p* = 0.015) on HS and Core Endurance, and a total indirect effect on LL Strength Asymmetries, through HS and Core Endurance (PC −0.077, *p* = 0.042) (Table 8). Consequently, HS Strength had a statistically significant total effect (PC −0.295, *p* = 0.008) on LL Strength Asymmetries (Table 7). Furthermore, the increase in previous injuries led to a decrease in HS and Core Endurance (PC −0.207, *p* = 0.029) and an increase in the reported burnout (ABQ) by the athletes (PC −0.262, *p* = 0.004).

On the other hand, contrary to our hypothesis, relationships showed a statistically significant direct relationship between HS Strength and the Number of New Non-contact LL Injuries, HS and Core Endurance with Number of New Non-contact LL Injuries, and ABQ with Number of New Non-contact LL Injuries. According to these results, stronger athletes with higher levels of strength and endurance and lower burnout tended to be more prone to injuries.

Overall, strength asymmetries and previous injuries had the most significant direct effect on the Number of New Non-contact LL Injuries. However, HS and Core Endurance seemed to play a significant role in the model, acting as mediating factor in the indirect effects of other factors such as HS Strength.

**Figure 7:**
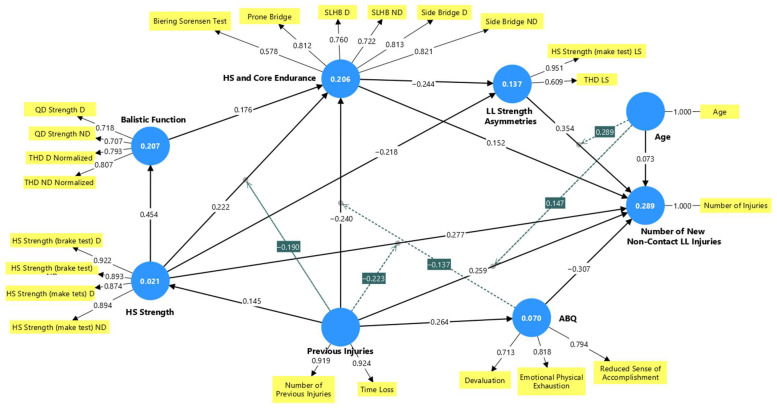
Model with the main moderating effects of age, Previous Injuries, and ABQ. Moderating effects were calculated as the average moderator effect presented in the simple association line plus the moderator effect for increased moderator levels and minus the moderating effect for decreased moderator level. For instance, in the association of LL Strength Asymmetries with Number of New Non-contact LE Injuries, the simple effect was 0.354. This association with an increased level of age moderating effect was calculated as 0.354 + 0.289, whereas the decreased level of age moderating effect was calculated as 0.354 − 0.289. Abbreviations: ABQ—athlete burnout questionnaire, HS—hamstring, QD—quadriceps, LL—lower limb, D—dominant, ND—non-dominant, THD—single-leg triple hop for distance, SLHB—single-leg hamstring bridge, LS—limb symmetry.

**Figure 8:**
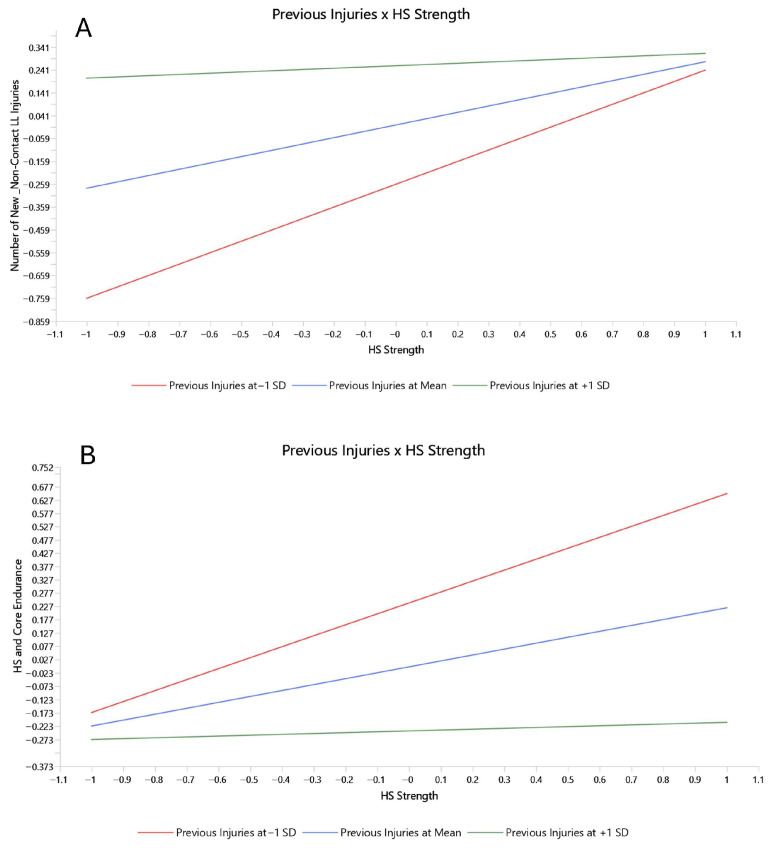
Simple slope analyses of the moderating effect of previous injuries. (**A**) Moderating effect of previous injuries on the relationship of HS strengh with number of new non-contact LL injuries; (**B**) Moderating effect of previous injuries on the relationship of HS strength with HS and core endurance. The green line represents the assotiation of the factors with 1 SD increase on the effect of the moderator, the blue line represents the assotiation with a mean effect of the moderator, and the red line represents the assotiation with 1 SD decrease on the moderator affect.

In the next step, the moderating effects of Previous Injuries, ABQ, and Age were explored as presented in Figure 7 and further analyzed using simple slope analyses presented in Figure 8. The model fit remained unchanged, and there were no significant alterations in measurement model indices. Regarding the structural model assessment, slight changes were observed in the R^2^ values of the latent factors representing ABQ, HS and Core Endurance, and the Number of New Non-contact LL Injuries. The exploratory power of the model, as indicated by the R^2^ values for the aforementioned factors, increased following the inclusion of the moderating effects. This partially proves the complexity of the factor’s interaction.

Previous Injuries exerted a significantly moderate effect on the relationships among neuromuscular factors and their influence on the Number of New Non-contact LE Injuries. In particular, as presented in Figure 8, the increased level of Previous Injuries (green line in Figure 8A) attenuated the positive association between HS Strength and the Number of New Non-contact LL Injuries. Similarly, Previous Injuries attenuated the significant positive association between HS Strength and HS and Core Endurance. The green line in Figure 8B shows the association between HS Strength with HS and Core Endurance under a high level of the moderating effect of Previous Injuries, in contrast to the red line that indicates a low level of the previous injury effect.

**Figure 9:**
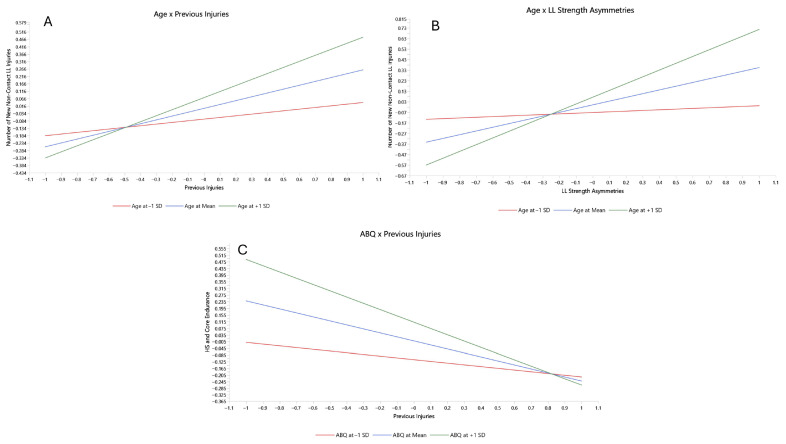
Simple slope analyses of the moderating effect of age and ABQ. (**A**) Age moderating effect on the relationships of previous injuries with new non-contact LL injuries; (**B**) Age moderating effect on the relationship of LL strength asymmetries with new non-contact LL injuries; (**C**) Moderating effect of ABQ on the relationship of previous injuries with HS and core endurance. The green line represents the assotiation of the factors with 1 SD increase on the effect of the moderator, the blue line represents the assotiation with a mean effect of the moderator, and the red line represents the assotiation with 1 SD decrease on the moderator affect.

Similarly, ABQ moderates the interaction between Previous Injuries and HS and Core Endurance (Figure 9C). Higher ABQ scores strengthened the negative relationships between Previous Injuries and the factor of HS and Core Endurance.

Further, the significant positive association between Previous Injuries and the Number of New Non-contact LL Injuries was strengthened by higher levels of moderating effect of age (Figure 9A). Additionally, greater age amplified the positive association between LL Strength Asymmetries and the Number of New Non-contact LL Injuries (Figure 9B). These results provide evidence that older athletes with a history of previous injuries and LL Strength Asymmetries are at higher risk of sustaining a non-contact LL injury.

## 4. Discussion

In the current investigation, a PLS-SEM analysis was carried out to explore the interrelationships among neuromuscular factors, Previous Injuries, athletes’ burnout, and age, with the incidence of non-contact LL football injuries recorded during one competitive season. The model identified the direct and indirect associations among the factors. The most significant findings were that LL Strength Asymmetries and previous injuries had a significant direct effect on the incidence of non-contact LL injuries. In addition, hamstring and core endurance, age, previous injuries, and athlete burnout (as measured by the ABQ) exerted important indirect and mediating effects within the aetiologic model. To the authors’ knowledge, this is the first study to employ a SEM approach to evaluate the complex interrelationships among intrinsic risk factors associated with football injury risk.

The incidence of non-contact LL injuries recorded in the current prospective study was 1.97 injuries per 1000 athlete-hours of exposure. In the present analysis, only time-loss non-contact LL injuries were considered; other contact injuries or non-time-loss overuse complaints were not included. These methodological choices led to a significantly lower injury incidence compared to those reported in the existing literature. Precisely, López-Valenciano et al. [2] reported an incidence of 8.1 injuries per 1000 h of exposure, while Fares et al. [1] reported an incidence of 11.14 per 1000 football hours in professional football players. In addition, another study [65] on professional football players revealed that total injury incidence ranges from 2.48 to 9.4 injuries per 1000 h of exposure. However, these studies report overall injury incidence, not limited to non-contact LL injuries as in our study. Moreover, previous epidemiological research on Greek amateur football players [66] reported non-contact LL injury incidence ranging from 0.87 to 2.61 injuries per 1000 h, depending on the playing position. These findings align closely with the results of the current study. The differences in competition level and the exclusive focus on non-contact LL injuries likely account for the observed variations between our findings and those in the broader literature.

The model showed that LL Strength Asymmetries have a statistically significant direct positive effect on prospectively recorded, non-contact LL injuries. This indicates that as strength asymmetries increase, the incidence of injury also increases. Additionally, the moderation analysis demonstrated that age amplifies this positive association between strength asymmetries and injury risk. Strength and functional inter-limb asymmetries are commonly measured variables in both research and clinical practice [67]. However, there is limited conclusive evidence regarding their association with non-contact LL injury risk [67,68]. The literature remains inconclusive, with some studies reporting a possible association between asymmetries and injury likelihood, while others report no significant relationship [9,67]. Notably, most studies have examined the isolated effect of strength asymmetries on injury rather than considering them within a multifactorial complex analytical framework. In addition, methodological inconsistencies across studies, such as differences in sample characteristics and asymmetry calculation methods, contribute to these conflicting findings [67,69]. The present investigation provides evidence that strength asymmetries exert a stronger influence on LL injury risk in older athletes. Moreover, although not demonstrated in the current analysis, other factors such as previous injury history and training workload may also moderate the relationship between strength asymmetries and injury risk [10,12]. Future research should further investigate these possible relationships.

Moreover, HS and Core Endurance, as well as LL strength factors, influence LL Strength Asymmetries. Specifically, HS and Core Endurance demonstrated a statistically significant direct negative associations with LL Strength Asymmetries. Additionally, HS and Core Endurance functioned as mediators in the statistically significant indirect relationship between LL strength and LL asymmetries. This indirect relationship stemmed from the direct positive effect of LL strength on HS and Core Endurance, indicating that athletes with higher LL strength tend to exhibit better muscular endurance. Overall, football players with higher scores in LL strength and HS and Core Endurance measures exhibited lower LL Strength Asymmetries. As a result of these associations, HS and Core Endurance exerted a near-significant (*p* = 0.067) indirect effect on the number of LL Injuries through LL Strength Asymmetries. To the authors’ knowledge, this is the first study to comprehensively model these interrelationships and their potential impact on non-contact LL injury. Previous research has explored the isolated relationships among strength, endurance, strength asymmetries, and performance [70,71,72] as well as the effect of these factors on injuries [9,54,72]. Future SEM analyses with larger sample sizes may provide more robust and more accurate insights into these complex interrelationships.

Furthermore, the PLS-SEM analysis revealed statistically significant associations between Previous Injuries and prospectively recorded non-contact LL injuries. This finding aligns with numerous studies identifying previous injuries as a strong risk factor that significantly increases the likelihood of subsequent injuries [9,23]. The previous literature has shown that previous injuries, such as hamstring strains and other severe LL injuries, are associated with various neuromuscular alterations that elevate the risk for recurrence [9,23,73]. In addition, the mediating and moderating relationships observed in the structural model offer further insights into these mechanisms. Previous Injuries demonstrated a significant moderating effect on neuromuscular characteristics and their associations. Specifically, Previous Injuries moderated the relationship between HS Strength and HS and Core Endurance, as well as the relationship between HS Strength and LL injuries. These findings indicate that previous injuries substantially influence neuromuscular interrelationships. Moreover, previous injuries were found to interact with burnout symptoms (ABQ). Particularly, previous injuries increased burnout symptoms, while burnout (ABQ) acted as moderator in the relationship between Previous Injuries and HS and Core Endurance. Higher ABQ scores strengthened the negative effect of previous injuries on HS and Core Endurance. While current study did not identify a direct association between ABQ scores and new injuries, in contrast with previous research [15,17], it supports the inclusion of psychological factors such as burnout within the injury etiology model, primarily through their interaction with previous injury history [74]. In line with prior studies [3,15,23], the findings suggest that neuromuscular adaptations and the presence of burnout following injuries affect athletes’ neuromuscular coordination, thereby increasing the risk of injury recurrence [3,15,23].

A surprising result of the PLS-SEM analysis was the direct relationship, contrary to our hypothesis, of HS Strength, HS and Core Endurance, and ABQ scores with prospectively recorded non-contact LL injuries. In particular, an increase in HS Strength demonstrates a statistically significant positive association with non-contact LL injuries, indicating that athletes with greater strength are more susceptible to such injuries. Similarly, a nearly statistically significant positive correlation was observed between HS and Core Endurance and non-contact LL injuries. Furthermore, the ABQ scores exhibited a negative relationship with non-contact LL injuries, suggesting that lower levels of burnout, as indicated by reduced ABQ scores, were associated with an increased non-contact LL injuries risk. As previously reported in the literature, we initially hypothesized that lower strength, endurance values, as well as higher burnout levels would increase injury risk [3,4,15]. However, the model showed the opposite trend [75]. Theoretically, these counterintuitive findings may be interpreted through a systems-thinking perspective [3]. Specifically, the model is limited to internal risk factors, excluding other important factors such as workload, which may exert a moderate effect on the observed associations [75]. A possible explanation of these unexpected results is that stronger, more fit, and psychologically well-prepared athletes are more frequently selected to participate in matches or training, leading to greater exposure to workload and external risk factors and, consequently, an increased risk of injury [3,4,12]. Additionally, moderation analysis revealed that Previous Injuries moderated the direct associations between HS Strength and LL injuries. Specifically, the model showed that as the level of previous injuries increased, the positive association between HS Strength and LL injuries was attenuated. Therefore, the inclusion of additional factors in the model, such as workload, along with larger sample sizes, may offer a more comprehensive understanding of these complex interrelationships. Consequently, this particular result should be interpreted with caution, especially when being applied to injury prevention and rehabilitation planning.

In addition, when the moderating effect was included, the R^2^ value increased from 0.210 to 0.286. This suggests that the risk of injury involves multiple indirect and moderating relationships, and the predictive power of the model improved accordingly. Nonetheless, the model incorporated only a subset of intrinsic risk factors, and the relatively low R^2^ values were expected. Several significant risk factors, such as the exposure rate and workload, were not included in the model [12]. Incorporating additional intrinsic and extrinsic variables may significantly enhance the explanatory power of future models.

The limitations of the quantitative PLS-SEM analysis primarily concern the small sample size and the limited number of factors assessed. Sample size requirements for PLS-SEM analysis depend on both model complexity and path coefficients. Although PLS-SEM is known to produce meaningful results with smaller samples compared to other SEM approaches, general guidelines recommend a minimum of 100–200 participants [26]. According to the “10-times rule”, the minimum required sample size should be ten times the number of arrows pointing to the most highly predicted factor [26]. In the current model, HS and Core Endurance were predicted by nine arrows, indicating a minimum of 90 participants. However, post hoc analysis revealed that to achieve α = 0.05 and 80% statistical power, more than 200 participants would be required for approximately half of the direct, indirect, and mediated paths. Therefore, the current sample of 97 athletes was relatively small, and future studies should aim for a sample size exceeding 250 participants. Sample size constraints may also explain the exclusion of flexibility and other asymmetry measures from the PLS-SEM analysis. While these variables are important for assessing injury risk, they did not meet the criteria required for construct validity, reliability, and variable loading and thus could not be included in any latent construct. Consequently, they were not evaluated in the current PLS-SEM model. These variables should be either assessed independently or incorporated into other PLS-SEM studies with larger sample sizes. Furthermore, the heterogeneity of the sample used in this study represents a limitation that warrants consideration. The sample included football players from different competitive levels (under 19, third division, second division), which may introduce variability in the workload across the season. Although the analysis controlled for age, and all teams followed a similar training and game schedule, the competitive level was not controlled, potentially influencing some of the observed relationships. Moreover, the PLS-SEM model focused exclusively on intrinsic risk factors derived from a field-based assessment protocol, thereby limiting the understanding of the full complexity of injury risk. Incorporating extrinsic factors, such as workload characteristics, would offer a comprehensive view of the multifactorial nature of risk factors. A notable limitation of prospective studies assessing intrinsic risk factors is their failure to account for players’ workload, which often acts as a significant confounding variable. As a result, the statistical models may unintentionally capture workload effects. Adding extrinsic variables such as workload, along with intrinsic laboratory-based biomechanical measures, would further improve the understanding of the non-linear and dynamic nature of injury risk.

For a practical point of view, exercise-based injury prevention programs should adopt a multifactorial approach. Based on the study’s results, prevention strategies should emphasize improving hamstring and core muscle endurance ability, reducing strength asymmetries, particularly in older athletes, and implementing individualized management strategies for older players and those with a history of previous injuries. Finally, preventing athletes’ burnout is equally important, as it appears to have a secondary yet meaningful effect within the model.

## 5. Conclusions

The application of the SEM approach in this study elucidates the multifactorial nature of non-contact LL injuries by clarifying the interrelationships among intrinsic risk factors. The model highlights the statistically significant positive associations between LL Strength Asymmetries, including HS isometric strength and THD asymmetries, and the incidence of Previous Injuries, with the frequency of non-contact LL injuries. In addition, age was found to significantly amplify the positive effect of LL Strength Asymmetries on non-contact LL injury risk. Moreover, HS and Core Endurance, Previous Injuries, and athlete burnout (ABQ) were shown to exert indirect and mediating effects in the model. However, further research is warranted, involving larger sample sizes and the inclusion of both intrinsic and extrinsic risk factors, to develop a more comprehensive understanding of the mechanism underlying specific types of injuries. Understanding the interdependencies among risk factors may enable researchers and medical practitioners to adopt a holistic, systems-based approach to injury risk management, thereby facilitating the development of more effective, evidence-based prevention strategies.

## Figures and Tables

**Table 1:** Sample characteristics.

Factors	Mean ± SD
Age	22.16 ± 5.03
Weight (kg)	74.44 ± 7.71
Height (cm)	178.82 ± 6.27
BMI	23.25 ± 1.76
Age started playing football	7.75 ± 2.94
Years playing in professional level	3.79 ± 4.06
Participation in matches the previous year	19.26 ± 9.48
Hours of training per day the previous year	2.33 ± 0.64
Days training per week the previous year	5.53 ± 0.60

**Table 2:** Preseason measurements.

	Measurements			
	Factors		Tests Description	Equipment/Variable
1	Anthropometric	Weight		Force platform (kg)
		Height		Measuring tape (cm)
		Leg length		Measuring tape (cm)
		Plantar length		Measuring tape (cm)
2	Flexibility	Hamstrings	Passive strain leg raise (supine position)	Inclinometer (°)
		Iliopsoas	Thomas test (supine position)	Qualitative assessment pass/no pass
		Rectus Femoris	Ely’s test (prone position)	Inclinometer (°)
		Hip internal/external rotation	Hip internal/external ROM (prone position)	Inclinometer (°)
		Ankle dorsiflexion	Weight-bearing lunge test (standing position)	Inclinometer (°)
3	Strength	Abductor	Side-lying position. Isometric test for approximately 5 s.	HHD (Nm/kg)
		Hamstrings (Brake)	Prone position. Break test after approximately 3 s isometric contraction; 30° knee flexion.	HHD (Nm/kg)
		Hamstring (Make)	Prone position 30° knee flexion. Isometric contraction for approximately 5 s.	HHD (Nm/kg)
		Quadriceps	Isometric contraction. Sitting position with the use of a stabilization belt.	HHD (Nm/kg)
4	Functional and ballistic performance	Hop distance	Single-leg triple hop for distance (THD) Test	Hop distance (cm), max distance divided by athlete’s height (normative value)
5	Endurance	Abdominal	Prone Bridging Test	Stopwatch (maximum time in second)
		Lateral abdominal	Side Bridging Test	Stopwatch (maximum time in second)
		Back muscle	Biering–Sorensen test	Stopwatch (maximum time in second)
		Hamstring	Single-leg hamstring bridge (SLHB).	Maximum repetitions

Abbreviations: kg—kilograms; cm—centimeter; Nm—newton meters; HHD—handheld dynamometer; s—seconds.

**Table 3:** Descriptive statistics of the preseason measurements for dominant and non-dominant lower limbs.

	Measured Variables	Min	Max	Mean	SD	Skewness	Kurtosis
ABQ	Emotional Physical Exhaustion	1.00	3.20	1.65	0.48	0.89	0.48
	Reduced Sense of Accomplishment	1.00	3.80	2.45	0.56	−0.37	0.39
	Devaluation	1.00	4.33	1.34	0.63	2.67	7.84
Dominant lower limb	Leg Length D	82.00	103.50	92.41	4.42	0.10	−0.06
	Flexibility WBLT D	24.00	51.50	37.04	5.43	0.22	−0.12
	Flexibility LSR D	53.00	96.50	77.18	8.66	−0.03	−0.14
	Flexibility Knee Flexion D	120.00	165.00	143.60	7.73	−0.08	0.85
	Flexibility Hip Internal D	11.00	53.00	29.32	7.86	0.33	0.24
	Flexibility Hip External D	27.50	75.50	47.46	8.94	0.33	0.65
	Flexibility Thomas Test D (1 = negative, 2 = positive)	1.00	2.00	1.67	0.47	−0.73	−1.49
	Strength Abductor D	1.68	3.02	2.28	0.28	0.00	−0.44
	Strength HS Isometric (brake test) D	1.12	2.37	1.61	0.24	0.53	0.34
	Strength HS Isometric (make test) D	1.00	2.21	1.49	0.21	0.47	0.98
	Strength Quadriceps D	1.85	4.30	3.04	0.46	0.01	−0.18
	THD D	2.43	4.29	3.23	0.31	0.28	0.97
	SLHB D	10.00	60.00	32.77	9.99	0.13	−0.10
Non-dominant lower limb	Leg Length ND	82.00	104.50	92.51	4.43	0.14	0.05
	Flexibility WBLT ND	23.00	55.00	37.47	5.62	0.03	0.23
	Flexibility LSR ND	51.50	97.00	78.71	8.74	−0.18	0.00
	Flexibility Knee Flexion ND	121.00	160.00	143.98	8.20	−0.40	0.40
	Flexibility Hip Internal ND	10.00	51.50	28.62	8.30	0.31	0.11
	Flexibility Hip External ND	26.00	67.50	48.73	8.89	−0.04	−0.52
	Flexibility Thomas Test ND (1 = negative, 2 = positive)	1.00	2.00	1.69	0.46	−0.84	−1.33
	Strength Abductor ND	1.34	2.99	2.19	0.28	−0.15	0.84
	Strength HS Isometric (brake test) ND	1.07	2.23	1.57	0.25	0.41	−0.17
	Strength HS Isometric (make test) ND	1.07	1.98	1.46	0.19	0.18	−0.40
	Strength Quadriceps ND	1.64	3.99	3.08	0.47	−0.25	−0.08
	THD ND	2.57	4.23	3.25	0.32	0.13	0.61
	SLHB ND	10.00	60.00	32.93	10.35	0.42	−0.38
Asymmetries	Flexibility WBLT LS	0.00	23.08	7.03	5.77	1.06	0.43
	Flexibility LSR LS	0.00	17.83	5.55	4.22	1.08	0.52
	Flexibility Knee Flexion LS	0.00	11.68	2.95	2.64	1.31	1.46
	Flexibility Hip Internal LS	0.00	50.00	15.28	10.47	0.83	0.84
	Flexibility Hip External LS	0.98	40.59	12.32	8.08	1.06	1.70
	Strength Abductors LS	0.00	33.76	8.12	6.33	0.94	1.47
	Strength HS Isometric (brake test) LS	0.00	21.01	6.90	5.16	0.68	−0.30
	Strength HS Isometric (make test) LS	0.14	21.29	5.65	4.43	1.10	1.17
	Strength Quadriceps LS	0.13	24.64	7.30	6.01	1.12	0.69
	THD LS	0.00	19.69	4.90	4.04	1.07	0.90
	SLHB LS	0.00	38.46	13.68	10.42	0.55	−0.55
Core endurance	Prone Bridge	49.00	380.00	175.40	76.12	0.84	0.11
	Side Bridge D	47.00	190.00	90.10	28.71	0.98	1.03
	Side Bridge ND	45.00	168.00	89.61	27.73	0.57	−0.55
	Biering–Sorensen Test	5.00	211.00	100.30	36.95	0.57	0.33

Abbreviations: ABQ—athlete burnout questionnaire, HS—hamstring, D—dominant, ND—non-dominant, THD—single-leg triple hop for distance, WBLT—weight-bearing lunge test, SLHB—single leg hamstring bridge.

**Table 4:** Characteristics of non-contact lower limb injuries recorded during the season.

Type of Injury	Frequency of Injuries (Injuries and Re-Injuries)	Re-Injuries	Percent (%)	Time Loss (Mean ± SD)	Injuries per 1000 Athlete-Hours Exposure
Sports hernia/abdominal tendinopathy	4	0	5.3	48.00 ± 30.53	0.10
Other lumbar/hip injury	5	0	6.7	8.00 ± 5.29	0.13
Groin strain	13	2	17.3	6.62 ± 6.25	0.34
Hamstring strain	16	2	21.3	13.31 ± 9.80	0.42
Quadriceps strain	12	1	16	13.00 ± 12.66	0.31
Unspecified thigh muscle strain	1	0	1.3	20.00 ± 0.00	0.03
Meniscus	1	0	1.3	60.00 ± 0.00	0.03
Other knee injury	5	0	6.7	15.00 ± 19.64	0.13
Lower leg injury	6	0	8	6.33 ± 4.85	0.16
Ankle injury	11	2	14.7	16.00 ± 20.99	0.29
Foot injuries	1	0	1.3	22.00 ± 0.00	0.03
Total	75	0	100		1.97

**Table 5:** PLS-SEM measurement model results for all non-contact lower limb injuries.

Latent Factors	Measured Indicators	Convergent Validity		Internal Consistency Reliability		Discriminant Validity	
		Loading	AVE	Cronbach’s Alpha	Reliability pA	Composite Reliability pc	HTMT
		>0.70	>0.50	0.60–0.90	0.60–0.90	0.60–0.90	<0.85
ABQ	Devaluation	0.753	0.606	0.674	0.676	0.822	YES
	Emotional Physical Exhaustion	0.818					
	Reduced Sense of Accomplishment	0.763					
LL Strength Asymmetries	HS isometric (make test) Strength LS	0.949	0.639	0.500	0.784	0.772	YES
	THD LS	0.614					
Ballistic Function	QD Isometric Strength D	0.719	0.574	0.755	0.770	0.843	YES
	QD Isometric Strength ND	0.707					
	THD D	0.792					
	THD ND	0.807					
HS Strength	HS Isometric (brake test) Strength D	0.922	0.802	0.919	0.932	0.942	YES
	HS Isometric (brake test) Strength ND	0.893					
	HS Isometric (make test) Strength D	0.874					
	HS Isometric (make test) Strength ND	0.894					
HS and Core Endurance	Biering–Sorensen Test	0.571	0.572	0.847	0.867	0.888	YES
	Prone Bridge	0.812					
	SLHB D	0.770					
	SLHB ND	0.734					
	Side Bridge D	0.807					
	Side Bridge ND	0.814					
Previous Injuries	Number of Previous Injuries	0.918	0.849	0.823	0.824	0.919	YES
	Time Loss	0.925					
Number of New Non-Contact LL Injuries	Number of injuries	1.000	1.000	1.000	1.000	1.000	YES

Abbreviations: ABQ—athlete burnout questionnaire, HS—hamstring, QD—quadriceps, LL—lower limb, D—dominant, ND—non-dominant, THD—single-leg triple hop for distance, SLHB—single-leg hamstring bridge.

**Table 6:** Results of directs effects.

Factors Interaction	Path Coefficients	T Values	*p* Values	95% Confidence Intervals (with Bias Correction)	f-Square
ABQ → Number of New Non-contact LL Injuries	−0.232	2.923	0.003 *	−0.368, −0.051	0.062
Ballistic Function → HS and Core Endurance	0.156	1.295	0.195	−0.102, 0.374	0.022
HS Strength → Ballistic Function	0.455	6.499	0.000 **	0.290, 0.577	0.260
HS Strength → HS and Core Endurance	0.248	2.428	0.015 *	0.015, 0.424	0.056
HS Strength → LL Strength Asymmetries	−0.218	1.862	0.063	−0.434, 0.025	0.050
HS Strength → Number of New Non-contact LL Injuries	0.243	2.199	0.028 *	0.012, 0.447	0.063
HS and Core Endurance → LL Strength Asymmetries	−0.243	2.504	0.012 *	−0.408, −0.015	0.063
HS and Core Endurance → Number of New Non-contact LL Injuries	0.200	1.701	0.089	−0.042, 0.417	0.041
LL Strength Asymmetries → Number of New Non-contact LL Injuries	0.293	2.920	0.004 *	0.077, 0.474	0.093
Previous Injuries → ABQ	0.262	2.896	0.004 *	0.044, 0.412	0.073
Previous Injuries → HS Strength	0.145	1.599	0.110	−0.044, 0.310	0.021
Previous Injuries → HS and Core Endurance	−0.207	2.182	0.029 *	−0.381, −0.008	0.049
Previous Injuries → Number of New Non-contact LL Injuries	0.233	2.442	0.015 *	0.038, 0.418	0.059

Abbreviations: ** *p* < 0.001, * *p* < 0.05, ABQ—athlete burnout questionnaire, HS—hamstring, LL—lower limb.

**Table 7:** Total Effects (direct and indirect).

Factors Interaction	Path Coefficients	T Values	*p* Values	95% Confidence Intervals (with Bias Correction)
ABQ → Number of New Non-contact LL Injuries	−0.232	2.923	0.003 *	−0.368, −0.051
Ballistic Function → HS and Core Endurance	0.156	1.295	0.195	−0.102, 0.374
Ballistic Function → LL Strength Asymmetries	−0.038	1.032	0.302	−0.126, 0.015
Ballistic Function → Number of New Non-contact LL Injuries	0.020	0.658	0.511	−0.014, 0.114
HS Strength → Ballistic Function	0.455	6.499	0.000 **	0.290, 0.577
HS Strength → HS and Core Endurance	0.319	4.069	0.000 **	0.138, 0.452
HS Strength → LL Strength Asymmetries	−0.295	2.645	0.008 *	−0.489, −0.049
HS Strength → Number of New Non-contact LL Injuries	0.221	2.160	0.031 *	0.001, 0.404
HS and Core Endurance → LL Strength Asymmetries	−0.243	2.504	0.012 *	−0.408, −0.015
HS and Core Endurance → Number of New Non-contact LE Injuries	0.129	1.086	0.278	−0.112, 0.349
LL Strength Asymmetries → Number of New Non-contact LL Injuries	0.293	2.920	0.004 *	0.077, 0.474
Previous Injuries → ABQ	0.262	2.896	0.004 *	0.044, 0.412
Previous Injuries → Ballistic Function	0.066	1.504	0.133	−0.021, 0.151
Previous Injuries → HS Strength	0.145	1.599	0.110	−0.044, 0.310
Previous Injuries → HS and Core Endurance	−0.161	1.527	0.127	−0.355, 0.056
Previous Injuries → LL Strength Asymmetries	0.007	0.154	0.877	−0.089, 0.102
Previous Injuries → Number of New Non-contact LL Injuries	0.178	2.284	0.022 *	0.018, 0.325

Abbreviations: ** *p* < 0.001, * *p* < 0.05, ABQ—athlete burnout questionnaire, HS—hamstring, LL—lower limb.

**Table 8:** Total indirect effects.

Factor Interaction	Indirect Effect	T Values	*p* Values	95% Confidence Intervals (with Bias Correction)
Ballistic Function → LL Strength Asymmetries	−0.038	1.032	0.302	−0.126, 0.015
Ballistic Function → Number of New Non-contact LL Injuries	0.020	0.658	0.511	−0.014, 0.114
HS Strength → HS and Core Endurance	0.071	1.216	0.224	−0.046, 0.185
HS Strength → LL Strength Asymmetries	−0.077	2.036	0.042 *	−0.157, −0.008
HS Strength → Number of New Non-contact LL Injuries	−0.023	0.407	0.684	−0.139, 0.079
HS and Core Endurance → Number of New Non-contact LL Injuries	−0.071	1.831	0.067	−0.163, −0.008
Previous Injuries → Ballistic Function	0.066	1.504	0.133	−0.021, 0.151
Previous Injuries → HS and Core Endurance	0.046	1.455	0.146	−0.012, 0.114
Previous Injuries → LL Strength Asymmetries	0.007	0.154	0.877	−0.089, 0.102
Previous Injuries → Number of New Non-contact LL Injuries	−0.055	1.052	0.293	−0.161, 0.045

Abbreviations: * *p* < 0.05, ABQ—athlete burnout questionnaire, HS—hamstring, LL—lower limb.

## Data Availability

The data supporting the findings of this study are available on reasonable request from the corresponding author. The data is not publicly available due to privacy and ethical restrictions.
